# Acetabular-only robot-assisted total hip arthroplasty is associated with lower surgeon stress index and perceived workload than conventional total hip arthroplasty: a prospective comparative cohort study

**DOI:** 10.1186/s42836-026-00442-2

**Published:** 2026-10-16

**Authors:** Muyang Yu, Mingwei Hu, Te Liu, Yiming Xu, Xingdong Yang, Huiming Peng, Xisheng Weng, Bin Feng

**Affiliations:** 1 Department of Orthopedic Surgery, Peking Union Medical College Hospital, Chinese Academy of Medical Sciences and Peking Union Medical College, Beijing, 100730 China; 2 State Key Laboratory of Complex Severe and Rare Diseases, Peking Union Medical College Hospital, Chinese Academy of Medical Sciences and Peking Union Medical College, Beijing, 100730 China

**Keywords:** Robot-assisted total hip arthroplasty, Surgeon workload, Physiologic stress, Heart rate variability, Surgery Task Load Index, Wearable sensors

## Abstract

**Background:**

Robot-assisted THA (RATHA) has been associated with greater component-positioning accuracy, but surgeon workload during RATHA remains unclear. This study compared objective physiologic stress and perceived workload during conventional THA (CTHA) and RATHA.

**Methods:**

This exploratory, hypothesis-generating study prospectively evaluated 60 elective primary THAs, comprising 30 CTHAs and 30 RATHAs, as repeated procedural observations from a single high-volume, fellowship-trained arthroplasty surgeon. All procedures were performed through a standardized posterolateral approach. RATHA used the MAKO system for acetabular preparation and cup implantation only. Intraoperative physiologic data were collected using a Hexoskin smart garment. Whole-case outcomes included heart rate, stress index, root mean square of successive differences (RMSSD), respiratory rate, minute ventilation, and energy expenditure rate. Perceived workload was assessed using the Surgery Task Load Index (SURG-TLX). Phase-specific stress index and RMSSD were analyzed across exposure, acetabular preparation, femoral preparation, and wound closure.

**Results:**

Baseline patient characteristics were comparable between groups. Operative time was significantly longer during RATHA than CTHA (141.50 ± 30.76 vs 116.86 ± 21.88 min, *p* < 0.001). Despite this, RATHA was associated with a lower SURG-TLX composite score (adjusted mean difference, − 0.47; 95% CI − 0.85 to − 0.09; *p* = 0.039), with lower scores for task complexity and situational stress. Whole-case stress index was lower during RATHA (adjusted mean difference, − 2.52; 95% CI − 4.21 to − 0.83; *p* = 0.004), whereas the between-group difference in whole-case RMSSD was not statistically significant (geometric mean ratio, 1.35; 95% CI 0.98–1.86; *p* = 0.067). Heart rate, respiratory rate, minute ventilation, and energy expenditure rate did not differ significantly between groups. Phase-specific analysis showed lower stress index during RATHA in exposure, acetabular preparation, and femoral preparation, but higher stress index during wound closure. RMSSD was higher during RATHA in exposure and acetabular preparation.

**Conclusions:**

In this single-surgeon cohort, the acetabular-only RATHA workflow was associated with lower perceived workload and a lower mean whole-case stress index than the CTHA workflow despite longer operative time. These hypothesis-generating findings warrant validation in multisurgeon studies.

## Background

Total hip arthroplasty (THA) is a high-volume, technically demanding procedure that places substantial physical and cognitive demands on the operating surgeon [1, 2]. Objective physiologic monitoring in arthroplasty has shown that, compared with total knee arthroplasty, THA can elicit higher surgeon cardiorespiratory strain and stress, and that revision arthroplasty, as a more complex form of arthroplasty, further amplifies these physiologic demands [3, 4]. This load is associated with a high prevalence of work-related musculoskeletal disorders and occupational injuries among orthopaedic surgeons, sometimes severe enough to require treatment or alter career trajectories [5, 6]. In addition, acute stress and fatigue are human-factors risks that can degrade surgical performance, decision-making, and non-technical skills [5, 7]. Therefore, from both occupational health and surgical performance perspectives, reducing surgeons’ intraoperative workload carries important clinical significance.

Robot-assisted THA (RATHA) has been increasingly adopted, with systematic reviews and meta-analyses reporting greater radiographic accuracy and, in some studies, lower complication rates than conventional manual THA (CTHA), although consistent superiority in patient-reported outcomes has not been demonstrated [8–10]. From the operator perspective, RATHA differs from CTHA through preoperative planning, intraoperative registration, system interaction, and real-time guidance. Learning-curve studies further indicate that efficiency and process stability vary with robotic experience [11, 12]. Whether these workflow differences are associated with distinct patterns of surgeon physiologic and perceived workload remains uncertain.

Wearable platforms such as the Hexoskin smart garment allow continuous intraoperative acquisition of electrocardiography- and respiration-derived metrics, enabling quantification of cardiorespiratory strain and physiologic stress under real operating-room conditions [3, 4]. Heart rate variability (HRV) in particular, has been supported by systematic review evidence as a feasible objective correlate of intraoperative mental stress when processed with standardized artifact correction and analysis workflows [13]. Meanwhile, perceived workload can be captured using the Surgery Task Load Index (SURG-TLX), a surgery-specific, multidimensional instrument that was developed and validated to assess surgeons’ perceived intraoperative task demands [14]. These advances enable comparisons of surgeons’ objective and perceived workload between RATHA and CTHA, and support phase-based analyses that localize physiologic stress and strain to specific operative tasks.

Accordingly, we conducted an exploratory, hypothesis-generating, single-surgeon prospective study to compare cardiorespiratory strain, HRV-derived physiologic stress, and perceived workload during RATHA versus CTHA and to characterize phase-specific workload patterns.

## Methods

### Study design

This exploratory, hypothesis-generating, prospective comparative cohort study was conducted at a tertiary academic practice between July 2025 and January 2026. The study protocol was approved by the institutional review board of the participating center (approval number: I-24PJ1047) and was conducted in accordance with the Declaration of Helsinki. Written informed consent was obtained from all included patients, and the participating surgeon provided consent for intraoperative physiologic monitoring and postoperative workload assessment. One high-volume, fellowship-trained arthroplasty surgeon participated in the study, with an annual case volume of approximately 70 CTHAs and 180 RATHAs.

### Surgeon demographics and workload measures

During each procedure, the participating surgeon wore a Hexoskin smart garment (Hexoskin, Montreal, QC, Canada), which continuously collected cardiorespiratory data throughout the operative period. The participating surgeon’s age, sex, height, weight, and body mass index were recorded and entered into the Hexoskin Smart software to facilitate physiologic output. For each case, the following physiologic parameters were extracted: heart rate (HR, beats per minute), stress index (sympathetic activation), heart rate variability (HRV, root mean square successive difference), respiratory rate (RR, breaths per minute), minute ventilation (MV, L/min), and energy expenditure rate (kcal/h). To enable phase-based analyses, start and end times for four predefined operative phases, including exposure, acetabular-side surgery, femoral-side surgery, and wound closure, were recorded in real time for each case.

Perceived workload was assessed using the SURG-TLX questionnaire. The participating surgeon completed the SURG-TLX once per case immediately after surgery within 10 min, providing a whole-case assessment of perceived workload for the overall procedure. SURG-TLX domain scores and the composite workload score were calculated according to the validated scoring approach.

### Radiographic and safety outcomes

Postoperative anteroposterior pelvic radiographs were evaluated for cup inclination, radiographic anteversion, Lewinnek safe-zone status, and absolute limb-length discrepancy (LLD). Cup inclination was measured relative to the interteardrop line, and anteversion was calculated using the Lewinnek method [15]. Absolute postoperative LLD was measured using the interteardrop line and corresponding lesser-trochanter landmarks [16]. Calculated total blood loss was determined from preoperative and postoperative day 3 hematocrit values using the Gross formula, with predicted blood volume calculated using the Nadler formula [17, 18]. Safety outcomes included perioperative transfusion, intraoperative complications, and complications, readmissions, reoperations, and revisions within 90 days.

### Case selection and data collection

Eligible cases included elective primary total hip arthroplasties performed between July 2025 and January 2026 using either RATHA with the MAKO system (Stryker, Mahwah, NJ, USA) or CTHA. Cases were eligible when the participating surgeon served as the primary operator. We excluded simultaneous bilateral THA and complex primary THA, including developmental dysplasia of the hip with Crowe type III/IV, substantial acetabular or proximal femoral deformity, severe bone loss requiring structural bone grafting or metal augmentation, hip ankylosis or severe contracture, and any history of prior ipsilateral hip surgery (e.g., osteotomy or internal fixation for fracture). Among cases meeting these clinical eligibility criteria, surgical technique was selected through shared decision-making between the patient and surgeon and was based primarily on patient preference. Acetabular morphology, diagnosis, and anticipated case complexity were not used as clinical criteria for selecting RATHA versus CTHA. Cases were further excluded if physiologic data were unusable because of inadequate signal quality or major recording interruptions.

Patient demographics (age, sex, and body mass index), surgical indication, laterality, American Society of Anesthesiologists (ASA) classification were collected for each included case.

### Surgical technique

All included procedures were scheduled as the first case of the operative day to minimize the potential influence of accumulated surgeon fatigue. All procedures were performed in the lateral decubitus position under general anesthesia through a standardized posterolateral approach. CTHA was performed using standard mechanical instrumentation, with acetabular and femoral preparation and component positioning guided by intraoperative anatomic landmarks and the surgeon’s routine technique. In the CTHA group, the acetabular component was a G7 cup and the femoral component was a Taperloc Complete primary femoral stem (Zimmer Biomet, Warsaw, IN, USA). In RATHA, robotic assistance was used for acetabular-side preparation and cup implantation only, whereas femoral preparation and stem implantation were completed using standard instrumentation. In the RATHA group, the acetabular component was a Trident cup and the femoral component was an Accolade II stem (Stryker, Mahwah, NJ, USA). Intraoperative fluoroscopy was not routinely used in either group. Because the implant and instrumentation systems were specific to each technique and were not standardized across groups, the comparison represents the complete CTHA and RATHA workflows rather than the isolated addition of robotic guidance.

To enable phase-based analyses, operative phase start and end times were recorded in real time for each case by the circulating nurse using a standardized data collection form. Exposure was defined from skin incision to the start of acetabular-side work. Acetabular preparation was defined from initiation of acetabular preparation to completion of liner implantation. Femoral preparation was defined from initiation of femoral preparation to completion of component assembly, reduction, and final assessment of stability and leg length. Wound closure was defined from initiation of closure to completion of skin closure. Total operative time was defined as skin incision to completion of wound closure.

### Statistical analyses

Given the absence of prior live clinical studies directly comparing wearable-derived surgeon workload between robot-assisted and conventional THA, this study was designed as an exploratory, prospective comparative feasibility cohort. The a priori enrollment target was informed by the closest available robotic arthroplasty workload literature. Haffar et al. included 20 procedures per group in a live clinical comparison of surgeon physiologic workload during robot-assisted and conventional TKA [19]. In a cadaveric comparison of robot-assisted and conventional direct anterior THA, Caba et al. reported substantial differences in relative physiologic stress and modified SURG-TLX physical and mental demand [20]. Collectively, these studies informed the selection of Cohen’s d = 0.8 as an indicative large-effect benchmark rather than a precisely estimated outcome-specific effect. An a priori calculation based on this benchmark indicated that 26 procedures per group would provide 80% power at a two-sided α of 0.05. Because the calculation addressed only a large standardized difference, it was used to set the feasibility cohort size and did not establish adequate power for smaller effects or broader confirmatory inference. After allowing for potential signal loss, recording interruption, or incomplete workload assessment, we planned to include 30 procedures per group, resulting in a total target sample size of 60. Because workload was assessed in a single surgeon, the surgeon was the experimental unit (*n* = 1), and individual procedures represented repeated within-operator observations. Accordingly, all analyses were interpreted as within-surgeon associations.

Continuous variables were summarized as mean (standard deviation) or median (interquartile range), as appropriate, and categorical variables as counts (percentages). Normality was assessed using Q-Q plots and the Shapiro–Wilk test. Between-group comparisons used independent-samples t tests or Mann–Whitney U tests for continuous variables and chi-square or Fisher’s exact tests for categorical variables, as appropriate.

For the SURG-TLX outcomes and whole-case physiologic outcomes, adjusted between-group differences were estimated using multivariable linear regression models that included surgical technique, patient age, sex, BMI, and ASA physical status. Age and BMI were entered as continuous variables, sex as a categorical variable, and ASA physical status as an ordinal variable. These preoperative covariates were selected a priori as potential indicators of case mix under the assumption that they could be associated with technique selection and case-related operative demands that may influence the surgeon’s physiologic responses. Adjustment was intended to reduce measured case-mix differences and was not considered sufficient to establish a causal effect of surgical technique. Adjusted mean differences with 95% confidence intervals were reported. The six SURG-TLX domain scores were treated as one family for multiplicity adjustment, and P values were adjusted using the Benjamini–Hochberg false discovery rate method. The composite score was analyzed separately, and its P value was not adjusted for multiplicity.

Heart rate variability processing was performed in Kubios HRV (version 4.2.0; Kubios Oy, Kuopio, Finland). Intraoperative heart rate variability was analyzed using a 5-min moving window with a 2.5-min step. The root mean square of successive differences (RMSSD) and stress index were selected as the primary heart rate variability-derived metrics. RMSSD values were summarized as geometric means across valid windows, whereas stress index values were summarized as arithmetic means across valid windows. RMSSD was analyzed on the natural logarithmic scale, with back-transformed values reported for interpretability. Because the moving windows were equally spaced, the whole-case summaries were implicitly weighted by the duration of each operative phase.

For the phase-specific analyses, stress index and RMSSD were compared between CTHA and RATHA across the four predefined operative phases using linear mixed-effects models. The models included surgical technique, operative phase, the technique-by-phase interaction, patient age, sex, BMI, and ordinal ASA physical status as fixed effects, with a random intercept for case to account for repeated phase-level measurements within the same procedure. RMSSD was modeled on the natural logarithmic scale. Phase-specific between-group contrasts were estimated from the fitted models and reported as adjusted mean differences for stress index and adjusted geometric mean ratios for RMSSD, each with a 95% confidence interval. For each outcome, P values for the four phase-specific comparisons were adjusted using the Benjamini–Hochberg false discovery rate method.

All tests were two-sided, and statistical significance was set at *p* < 0.05. Statistical analyses were performed using R software (version 4.5.0; R Foundation for Statistical Computing, Vienna, Austria).

## Results

### Study population and baseline characteristics

**Fig. 1:**
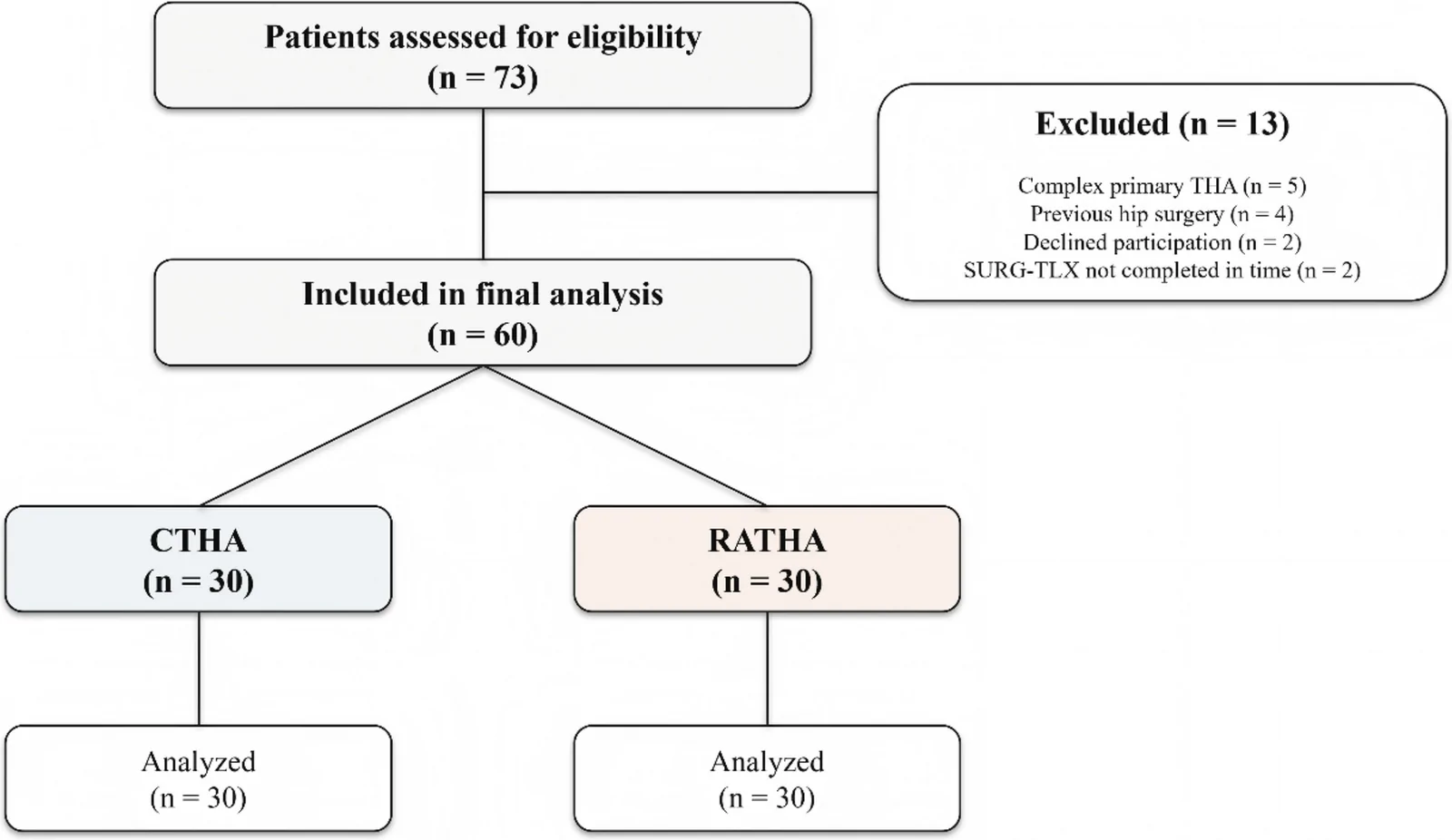
Study flow diagram. CTHA, conventional total hip arthroplasty; RATHA, robot-assisted total hip arthroplasty; SURG-TLX, Surgery Task Load Index

A total of 73 patients were assessed for eligibility. Thirteen patients were excluded because of complex primary THA (*n* = 5), previous hip surgery (*n* = 4), declined participation (*n* = 2), or SURG-TLX not completed in time (*n* = 2). The final analysis included 60 patients, with 30 patients in the CTHA group and 30 patients in the RATHA group (Fig. 1).

Baseline patient and surgical characteristics are summarized in Table 1. The two groups were comparable with respect to age, BMI, sex distribution, laterality, ASA classification, and diagnosis (all *p* > 0.05). Operative time was significantly longer in the RATHA group than in the CTHA group (141.50 ± 30.76 min vs 116.86 ± 21.88 min, *p* < 0.001).

**Table 1:** Baseline patient and operative characteristics

Variable	CTHA (*n* = 30)	RATHA (*n* = 30)	*p*-value
Age (yrs)	56.43 ± 11.65	58.21 ± 13.05	0.578
BMI (kg/m^2^)	24.42 ± 3.32	26.01 ± 3.30	0.068
Operative time (min)	116.86 ± 21.88	141.50 ± 30.76	< 0.001
Sex			0.438
Men	17 (56.7%)	14 (46.7%)	
Women	13 (43.3%)	16 (53.3%)	
Laterality			1.000
Left	13 (43.3%)	13 (43.3%)	
Right	17 (56.7%)	17 (56.7%)	
ASA classification			0.610
1	8 (26.7%)	5 (16.7%)	
2	20 (66.7%)	22 (73.3%)	
3	2 (6.7%)	3 (10.0%)	
Diagnosis			0.432
ONFH	17 (56.7%)	12 (40.0%)	
DDH	11 (36.7%)	15 (50.0%)	
HOA	2 (6.7%)	3 (10.0%)	

*Note:* Values are presented as mean ± SD or *n* (%)

*Abbreviations:* CTHA, conventional total hip arthroplasty; RATHA, robot-assisted total hip arthroplasty; BMI, body mass index; ASA, American Society of Anesthesiologists; ONFH, osteonecrosis of the femoral head; DDH, developmental dysplasia of the hip; HOA, hip osteoarthritis

### SURG-TLX workload scores

Subjective workload, assessed using the SURG-TLX, differed between groups in the composite score and selected domains (Table 2). The composite SURG-TLX score was significantly lower in the RATHA group than in the CTHA group (adjusted mean difference, − 0.47; 95% CI − 0.85 to − 0.09; *p* = 0.039).

**Table 2:** SURG-TLX workload scores by surgical technique

Variable	CTHA (*n* = 30)	RATHA (*n* = 30)	Adjusted mean difference (95% CI)	*p*-value ^b^
Mental Demands	6.93 ± 1.05	6.50 ± 0.55	− 0.47 (− 0.92 to − 0.03)	0.067
Physical Demands	6.79 [5.51, 8.03]	6.29 [5.90, 6.81]	− 0.32 (− 0.92 to 0.28)	0.336
Temporal Demands	4.86 ± 1.18	5.18 ± 1.28	0.34 (− 0.37 to 1.04)	0.342
Task Complexity	6.75 [5.65, 7.56]	4.53 [4.12, 5.84]	− 1.45 (− 2.04 to − 0.87)	< 0.001
Situational Stress	4.50 [3.85, 6.38]	3.29 [2.51, 4.49]	− 1.18 (− 1.85 to − 0.51)	0.003
Distractions	1.50 [1.37, 2.03]	1.51 [1.18, 2.53]	0.28 (− 0.08 to 0.63)	0.170
Composite Score ^**a**^	5.30 ± 0.81	4.72 ± 0.59	− 0.47 (− 0.85 to − 0.09)	0.039

*Note:* Values in the CTHA and RATHA columns are unadjusted descriptive statistics and are presented as mean ± SD or median [IQR], as appropriate. Adjusted mean differences represent RATHA minus CTHA and were estimated using linear regression models adjusted for age, sex, BMI, and ASA physical status

^**a**^Composite score was calculated as the unweighted mean of the six SURG-TLX domains

^**b**^*p*-values for the six SURG-TLX domain scores were FDR-adjusted using the Benjamini–Hochberg method; the *p* value for the composite score was unadjusted

*Abbreviations:* CTHA, conventional total hip arthroplasty; RATHA, robot-assisted total hip arthroplasty; SURG-TLX, Surgery Task Load Index

Among the individual SURG-TLX domains, task complexity was lower in the RATHA group than in the CTHA group (adjusted mean difference, − 1.45; 95% CI − 2.04 to − 0.87; adjusted *p* < 0.001), as was situational stress (adjusted mean difference, − 1.18; 95% CI − 1.85 to − 0.51; adjusted *p* = 0.003). Mental demands showed a trend toward lower scores in the RATHA group (adjusted mean difference, − 0.47; 95% CI − 0.92 to − 0.03; adjusted *p* = 0.067), whereas physical demands, temporal demands, and distractions did not differ significantly between groups.

### Whole-case physiologic outcomes

Whole-case physiologic outcomes are shown in Table 3. Whole-case stress index was lower in the RATHA group than in the CTHA group (adjusted mean difference, − 2.52; 95% CI − 4.21 to − 0.83; *p* = 0.004). Although the geometric mean RMSSD was numerically higher during RATHA, the adjusted between-group difference was not statistically significant (geometric mean ratio, 1.35; 95% CI 0.98–1.86; *p* = 0.067). No significant between-group differences were observed in heart rate, respiratory rate, minute ventilation, or energy expenditure rate (*p* > 0.05).

**Table 3:** Whole-case physiologic outcomes by surgical technique

Variable	CTHA (*n* = 30)	RATHA (*n* = 30)	Adjusted mean difference (95% CI)	*p*-value
Heart rate (beats/min)	84.50 ± 5.36	82.93 ± 4.25	− 1.28 (− 3.90 to 1.33)	0.330
Stress index	12.84 ± 3.18	10.68 ± 3.26	− 2.52 (− 4.21 to − 0.83)	0.004
RMSSD^a^ (ms, geometric mean 95% CI)	31.39 (25.08, 39.28)	45.29 (36.78, 55.77)	1.35 (0.98 to 1.86)	0.067
Respiratory rate (breaths/min)	19.79 ± 2.71	19.36 ± 2.36	− 0.58 (− 2.02 to 0.87)	0.426
Minute ventilation (L/min)	22.76 ± 4.61	21.86 ± 3.81	− 1.89 (− 4.06 to 0.28)	0.087
Energy expenditure rate (kcal/h)	171.85 ± 30.31	175.65 ± 32.49	− 0.08 (− 17.78 to 17.62)	0.993

*Note:* Values in the CTHA and RATHA columns are unadjusted descriptive statistics and are presented as mean ± SD unless otherwise indicated. Adjusted mean differences represent RATHA minus CTHA and were estimated using linear regression models adjusted for age, sex, BMI, and ASA physical status. Except for RMSSD, estimates represent mean differences between RATHA and CTHA

^a^RMSSD was analyzed on the natural logarithmic scale. Group values are geometric means (95% CIs), and the adjusted estimate is the geometric mean ratio for RATHA versus CTHA

*Abbreviations:* CTHA, conventional total hip arthroplasty; RATHA, robot-assisted total hip arthroplasty; RMSSD, root mean square of successive differences

### Phase-specific Stress Index and RMSSD

**Fig. 2:**
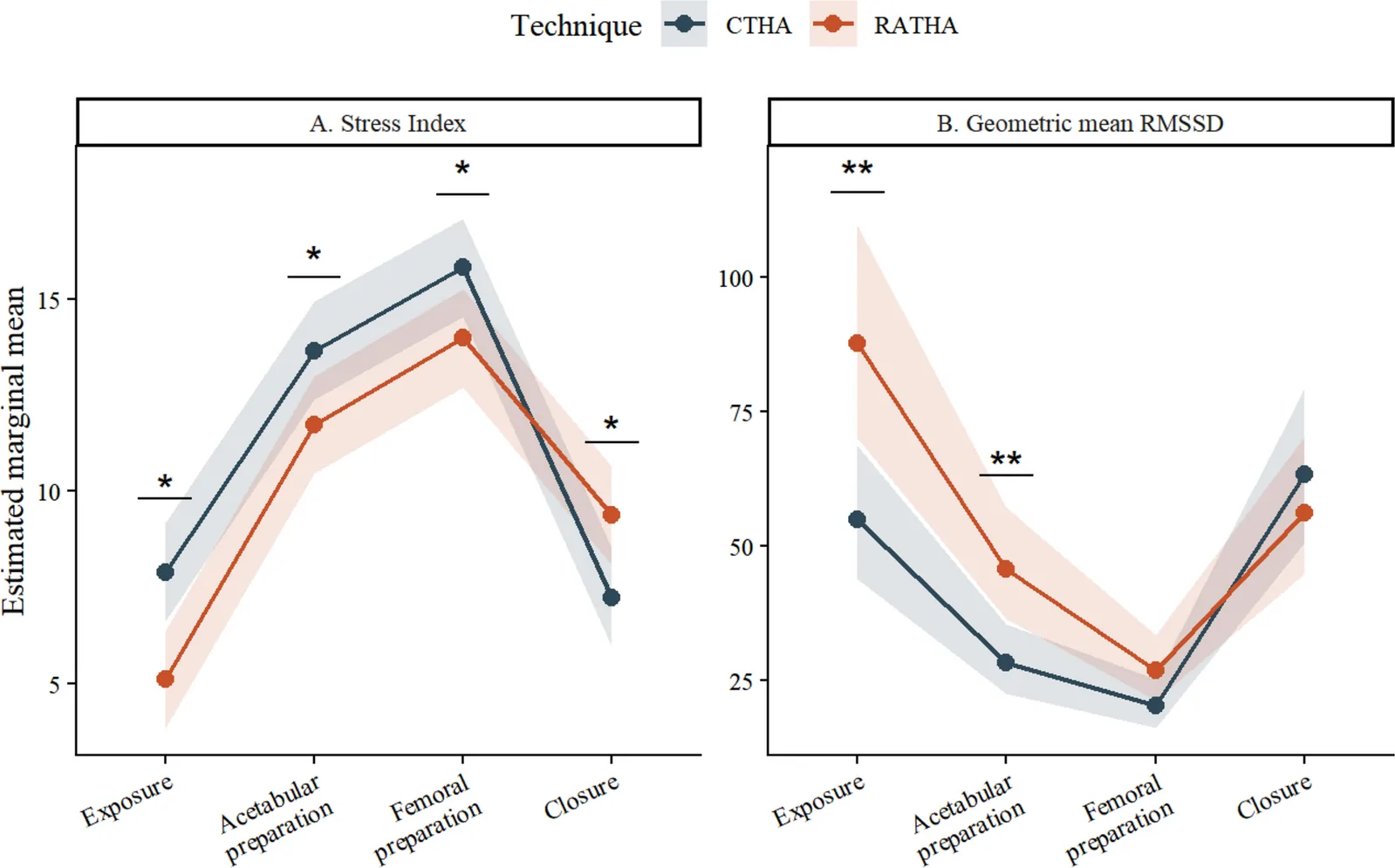
Phase-specific estimated marginal means of Stress Index and RMSSD by surgical technique. Panel A shows Stress Index across operative phases, and Panel B shows geometric mean RMSSD. Shaded areas indicate 95% confidence intervals. Asterisks indicate FDR-adjusted between-group differences within each phase. *FDR-adjusted *p* < 0.05; **FDR-adjusted *p* < 0.01; ***FDR-adjusted *p* < 0.001. CTHA, conventional total hip arthroplasty; RATHA, robot-assisted total hip arthroplasty; RMSSD, root mean square of successive differences

The phase-specific analysis showed that the physiologic workload pattern differed between CTHA and RATHA across operative phases (Table 4 and Fig. 2). In mixed-effects models adjusted for age, sex, BMI, and ASA, with random intercepts for case, RATHA was associated with lower stress index than CTHA during exposure (mean difference, − 2.78; 95% CI − 4.59 to − 0.97; adjusted *p* = 0.011), acetabular preparation (mean difference, − 1.92; 95% CI − 3.74 to − 0.11; adjusted *p* = 0.046), and femoral preparation (mean difference, − 1.84; 95% CI − 3.65 to − 0.03; adjusted *p* = 0.046). Conversely, during wound closure, stress index was higher in the RATHA group than in the CTHA group (mean difference, 2.14; 95% CI 0.32–3.95; adjusted *p* = 0.042).

**Table 4:** Phase-specific between-group differences in Stress Index and RMSSD

Outcome	Phase	CTHA (*n* = 30)	RATHA (*n* = 30)	Adjusted mean difference (95% CI)	Adjusted *p*-value ^c^
Stress Index ^a^	Exposure	8.13 ± 3.08	4.86 ± 1.22	− 2.78 (− 4.59 to − 0.97)	0.011
	Acetabular preparation	13.89 ± 2.52	11.48 ± 5.00	− 1.92 (− 3.74 to − 0.11)	0.046
	Femoral preparation	16.06 ± 4.00	13.73 ± 4.26	− 1.84 (− 3.65 to − 0.03)	0.046
	Wound closure	7.47 ± 2.20	9.12 ± 4.75	2.14 (0.32 to 3.95)	0.042
RMSSD ^b^	Exposure	52.60 (39.57 to 69.91)	91.02 (85.13 to 97.32)	1.60 (1.16 to 2.20)	0.009
	Acetabular preparation	27.07 (21.87 to 33.50)	47.37 (37.26 to 60.21)	1.62 (1.17 to 2.23)	0.009
	Femoral preparation	19.35 (15.51 to 24.13)	27.78 (21.62 to 35.68)	1.33 (0.96 to 1.83)	0.113
	Wound closure	60.68 (50.48 to 72.94)	58.27 (41.55 to 81.71)	0.89 (0.64 to 1.22)	0.459

Note: Values in the CTHA and RATHA columns are unadjusted descriptive statistics, presented as mean ± SD for Stress Index and geometric mean (95% CI) for RMSSD. Adjusted mean differences represent RATHA minus CTHA and were estimated using linear mixed-effects models adjusted for age, sex, BMI, and ASA physical status

^a^For Stress Index, adjusted mean differences represent mean differences calculated as RATHA minus CTHA

^b^RMSSD was analyzed on the natural logarithmic scale; adjusted mean differences represent geometric mean ratios for RATHA versus CTHA

^c^P values were adjusted using the Benjamini–Hochberg false discovery rate method

*Abbreviations:* ASA, American Society of Anesthesiologists; BMI, body mass index; CI, confidence interval; CTHA, conventional total hip arthroplasty; FDR, false discovery rate; RATHA, robot-assisted total hip arthroplasty; RMSSD, root mean square of successive differences; SD, standard deviation

For RMSSD, back-transformed geometric mean ratios indicated higher RMSSD in the RATHA group during exposure (ratio, 1.60; 95% CI 1.16–2.20; adjusted *p* = 0.009) and acetabular preparation (ratio, 1.62; 95% CI 1.17–2.23; adjusted *p* = 0.009). Between-group differences in RMSSD were not statistically significant during femoral preparation or wound closure.

### Radiographic and safety outcomes

Radiographic and safety outcomes are summarized in Table 5. Cup inclination, cup anteversion, and the proportion of cups within the Lewinnek safe zone did not differ between groups. Absolute postoperative LLD was lower in the RATHA group than in the CTHA group (2.9 ± 1.8 mm vs 4.2 ± 1.9 mm, *p* = 0.011). Calculated total blood loss did not differ between groups (803 ± 251 mL vs 878 ± 396 mL, *p* = 0.388). No patient in either group received a perioperative transfusion or experienced an intraoperative complication. Ninety-day follow-up was complete for all included patients, and no 90-day complications, readmissions, reoperations, or revisions were observed.

**Table 5:** Radiographic and safety outcomes

Outcome	CTHA (*n* = 30)	RATHA (*n* = 30)	*p*-value
Radiographic outcomes			
Cup inclination, degrees	40.7 ± 5.3	41.8 ± 1.5	0.268
Cup anteversion, degrees	22.5 ± 2.8	21.8 ± 2.3	0.271
Within Lewinnek safe zone ^a^	26 (86.7%)	28 (93.3%)	0.671
Absolute postoperative LLD, mm	4.2 ± 1.9	2.9 ± 1.8	0.011
Perioperative and safety outcomes			
Calculated total blood loss, mL	878 ± 396	803 ± 251	0.388
Transfusion	0	0	-
Any intraoperative complication ^b^	0	0	-
Any 90-day complication^c^	0	0	-
90-day readmission	0	0	-
90-day reoperation or revision	0	0	-

*Note:* Values are presented as mean ± SD or *n* (%)

^a^The Lewinnek safe zone was defined as cup inclination of 30° to 50° and anteversion of 5° to 25°

^b^Intraoperative complications included fracture, major soft-tissue or neurovascular injury, unplanned component revision, and robot-related adverse events or workflow conversion

^c^Ninety-day complications included dislocation, surgical-site or periprosthetic joint infection, symptomatic venous thromboembolism, periprosthetic fracture, new neurologic deficit, and death

*Abbreviations:* CTHA, conventional total hip arthroplasty; LLD, limb-length discrepancy; RATHA, robot-assisted total hip arthroplasty

## Discussion

This exploratory, hypothesis-generating, single-surgeon study found that, across repeated procedures performed by one high-volume operator, acetabular-only RATHA was associated with a lower whole-case stress index and lower perceived workload than CTHA despite longer operative time. Whole-case RMSSD, heart rate, ventilatory parameters, and energy expenditure rate did not differ significantly between techniques. To our knowledge, this study is among the first to combine wearable-derived physiologic monitoring with a surgery-specific workload instrument during live primary THA. Phase-specific analyses further showed that the observed workload pattern varied across operative phases.

The between-technique difference in the composite SURG-TLX score was concentrated in task complexity and situational stress, whereas physical demand, temporal demand, and distractions did not differ. In CTHA, acetabular preparation and cup implantation depend largely on freehand or mechanical-guide techniques, requiring integration of anatomical landmarks, pelvic orientation, and subjective estimation of cup position [21, 22]. By contrast, RATHA incorporates preoperative three-dimensional planning, intraoperative registration, haptic or visual guidance, and real-time feedback [23–25]. These workflow differences provide context for the observed associations, although the present study did not determine whether any specific feature accounted for the lower task-complexity or situational-stress scores during RATHA. However, the surgeon could not be blinded to surgical technique, creating potential expectation bias in the SURG-TLX assessments. Prior familiarity with and expectations regarding robotic assistance may have influenced ratings of task complexity and situational stress. The accompanying lower stress index was objectively recorded and not subject to questionnaire response bias, making differential reporting an unlikely sole explanation for the SURG-TLX differences. Nevertheless, expectations may also influence autonomic responses, and the extent to which the concordant subjective and physiologic patterns reflect workflow-related differences versus expectation-related psychophysiologic effects cannot be determined. The absence of a difference in physical demand is also consistent with the shared surgical approach, positioning, manual exposure, manual femoral preparation, and standard closure. Taken together, this pattern suggests that, in this operator, RATHA was associated primarily with lower perceived cognitive and stress-related workload rather than with uniformly lower workload across all domains.

The whole-case physiologic findings provided partial objective context for the perceived workload results. RATHA was associated with a lower stress index despite longer operative time. Thus, operative duration and whole-case stress index were not directionally aligned in this operator. RATHA also included additional time for registration, verification, and other technology-mediated workflow steps [24, 26, 27]. Stress index and RMSSD are HRV-derived metrics reflecting different aspects of autonomic regulation [13, 28]. In contrast, heart rate and ventilation are influenced by a broader mixture of physical exertion, posture, respiration, ambient environment, and individual physiologic baseline. The present findings showed a lower whole-case stress index without corresponding differences in RMSSD, gross cardiorespiratory measures, or energy expenditure rate. This pattern suggests that, in this operator, the surgeon-centered differences associated with RATHA were concentrated in the HRV-derived stress index, perceived task complexity, and situational stress, rather than reflecting a generalized difference in autonomic state or in overall physical or metabolic demand.

The phase-specific analysis showed that the association between surgical technique and HRV-derived stress varied across the operation. During acetabular preparation, which was the phase in which robotic assistance was used, RATHA was associated with a lower stress index and higher RMSSD. The acetabular workflow in RATHA is informed by preoperative planning and includes registration and real-time spatial guidance. These features provide context for the observed association, although the present study could not determine whether any individual component accounted for it [29]. By contrast, exposure and femoral preparation were performed manually in both groups. Although acetabular reconstruction informs the subsequent assessment of combined anteversion, stability, and offset [30], any association between the acetabular robotic workflow and workload during femoral preparation remains hypothetical. Interpretation of the femoral-preparation finding is further limited by the use of different femoral implants and instrumentation, because the observed association may also be related to differences in instrument handling and preparation steps. Accordingly, the findings during exposure and femoral preparation should be regarded as unconfirmed secondary findings that cannot be attributed specifically to robotic guidance. Systems incorporating robotic femoral preparation may produce a different workload profile from the acetabular-only workflow studied here. Robot-guided femoral milling may reduce manual broaching and its associated physical demands, whereas femoral registration, execution monitoring, and troubleshooting may introduce additional cognitive and attentional demands during the femoral phase [12, 31]. The unconfirmed lower femoral-phase stress index observed in the present study should therefore not be extrapolated to platforms incorporating robotic femoral preparation. Direct comparative workload data across robotic THA workflows are lacking, and future studies should distinguish the direct workload effects of femoral robotic guidance from any downstream influence of acetabular planning on subsequent manual steps. The higher stress index during wound closure, an identical manual phase in both groups, may reflect its later occurrence within the longer RATHA procedures, cumulative fatigue, or end-of-case time pressure rather than the robotic step itself. Because whole-case HRV metrics were summarized across equally spaced moving windows, longer phases contributed more heavily to the overall estimates, which represent time-weighted averages of autonomic state rather than cumulative physiologic burden. The coexistence of a lower whole-case stress index with a higher closure-phase stress index is therefore not necessarily contradictory. It indicates that the RATHA workflow was not associated with lower workload throughout all operative phases and that the higher closure-phase stress index may relate to longer operative duration, cumulative fatigue, or end-of-case time pressure. Accordingly, the whole-case findings should be interpreted as average associations across the procedure rather than evidence of uniformly or cumulatively lower stress.

The radiographic and safety outcomes provide patient-level clinical context for the surgeon-workload findings. Previous studies have shown that robot-assisted THA can improve the precision of acetabular component positioning and may reduce postoperative LLD, although the reported effects on limb-length restoration have not been uniform across studies [32–35]. In the present cohort, cup inclination, cup anteversion, and Lewinnek safe-zone rates did not differ between groups, whereas absolute postoperative LLD was 1.3 mm lower with RATHA. This difference cannot be attributed specifically to robotic guidance because robotic assistance was limited to the acetabular side, femoral preparation remained manual, and the groups received different femoral implants and instrumentation. Moreover, both groups had a mean absolute LLD of less than 5 mm, and the clinical relevance of the observed between-group difference cannot be determined without patient-reported assessments of perceived limb-length inequality or postoperative function. Lewinnek safe-zone status should likewise be regarded as a radiographic descriptor rather than a definitive indicator of stability, because dislocation is multifactorial and may occur despite cup positioning within the traditional safe zone [36, 37]. Calculated total blood loss did not differ, and no perioperative transfusions, intraoperative complications, or 90-day adverse events were observed. However, the primary objective and sample-size planning of this study concerned surgeon workload rather than patient-level clinical outcomes. The resulting estimates for radiographic, blood-loss, and safety outcomes therefore remain imprecise, particularly for infrequent complications, and should not be interpreted as establishing equivalence between techniques or as evidence that lower surgeon workload improves clinical performance.

These findings extend the growing literature showing that surgeon physiologic workload in arthroplasty is sensitive to procedure type, revision complexity, surgical approach, and enabling technology. Prior studies have shown that surgeon stress and strain vary according to procedure type, revision complexity, and surgical approach. Khan et al. reported greater cardiorespiratory strain during THA than TKA, and found higher physiologic stress during revision than primary arthroplasty [3, 4]. Cozzarelli et al. demonstrated higher stress and strain with the direct anterior approach than with the posterior approach [38]. Together, these studies indicate that surgeon workload is not a fixed attribute of arthroplasty, but is sensitive to operative context. The present study extends this literature by examining whether surgeon workload differs between conventional and acetabular-only robot-assisted workflows during primary THA. Consistent with the robotic TKA findings of Haffar et al., RATHA was associated with lower whole-case stress index [19]. The present study also addresses an important gap in the robotic THA workload literature. Caba et al. reported lower physical and mental demand during robot-assisted direct anterior THA in a cadaveric setting, providing early evidence that robotic assistance may reduce surgeon workload [20]. However, cadaveric models, limited sample size, and smartwatch-based monitoring limit direct translation to live operative practice. The present study extends this literature by evaluating live primary THA using continuous Hexoskin-based monitoring, HRV processing, and phase-specific mixed-effects modeling across both groups. By incorporating surgeon-centered workload measures, this study adds another dimension to the conventional evaluation of RATHA based on component-positioning accuracy and patient-reported outcomes [8], although it did not determine whether the observed workload differences were associated with clinical performance or patient outcomes.

These findings have practical implications for technology assessment in arthroplasty. Robotic assistance is usually evaluated through component positioning, radiographic outliers, complications, operative time, and patient-reported outcomes [8, 9, 39]. Surgeon-centered outcomes deserve similar attention. Arthroplasty surgeons have a high prevalence of work-related musculoskeletal symptoms and occupational injuries, and adult reconstruction has been described as a high-risk subspecialty for work-related injury [40–42]. Observed differences in physiologic and perceived workload may be relevant to occupational sustainability, operative scheduling, training, and workflow optimization [1, 19, 20]. At the same time, a surgeon-centered benefit should not be assumed from technology adoption alone. Robotic workflows also include potential stressors related to setup, registration, troubleshooting, team coordination, and longer operative time [43–45]. A balanced evaluation should therefore consider accuracy, efficiency, cost, team workload, ergonomics, and surgeon physiologic burden together.

The principal limitation of this study is its single-surgeon design. The operator-level experimental unit was one surgeon, and the 60 procedures represented repeated observations within that individual rather than independent observations across surgeons. The participating surgeon was a high-volume arthroplasty surgeon performing approximately 180 RATHA procedures annually and was therefore well beyond the early robotic learning curve. Previous studies have demonstrated learning effects in operative efficiency and surgical-team confidence during the adoption of robotic THA [11, 46]. For novice robotic surgeons, registration, system calibration, and intraoperative troubleshooting may impose additional cognitive and technical demands that could attenuate or even reverse the workload pattern observed in this experienced operator. Furthermore, the HRV-derived responses and SURG-TLX ratings reflect this individual surgeon’s autonomic reactivity and perception of task burden. The findings are therefore hypothesis-generating within-operator observations and cannot be generalized to early-learning surgeons, lower-volume surgeons, trainees, or surgeons as a group without multisurgeon validation across different levels of robotic experience.

Several additional limitations should be acknowledged. First, the study was not randomized, and technique selection was made through shared decision-making and primarily reflected patient preference rather than predefined clinical triage criteria. Although measured baseline characteristics were comparable and complex primary cases were excluded, residual confounding by unmeasured factors, including subtle pelvic or acetabular morphology, may have influenced operative complexity and surgeon workload. The adjusted between-group findings should therefore be interpreted as associations rather than causal effects. Second, preoperative physiologic measurements were not obtained before each procedure. Consequently, the analyses could not account for day-to-day variation in the surgeon’s baseline autonomic state or evaluate within-case changes from baseline. The observed HRV differences should therefore be interpreted as adjusted between-workflow associations during surgery rather than changes attributable specifically to either workflow. Third, because the surgeon could not be blinded to surgical technique, expectation-related influences on both subjective SURG-TLX ratings and objectively recorded autonomic responses cannot be excluded. Fourth, because implant and instrumentation systems differed between groups, the specific contribution of robotic guidance could not be isolated from the broader device-specific workflow. Fifth, robotic assistance was limited to the acetabular side; therefore, the findings should not be generalized to fully robotic systems or platforms with femoral-side robotic guidance. Sixth, although the physiologic data provided objective measures relevant to workload, the study did not include direct ergonomic motion tracking, surgeon posture analysis, electromyography, or hand-force measurements. Consequently, the reasons for the absence of between-group differences in energy expenditure rate and perceived physical demand could not be fully characterized. Seventh, SURG-TLX was not collected separately for each operative phase, limiting direct alignment between subjective workload and phase-specific physiologic changes. Eighth, because whole-case HRV summaries were weighted by phase duration, they characterize average autonomic state and cannot determine whether cumulative physiologic burden differed between workflows. Finally, the sample size was determined for surgeon-workload outcomes and provided limited precision for the radiographic and safety outcomes, particularly infrequent complications. PROMs were not collected, precluding assessment of whether the observed workload and radiographic findings were accompanied by differences in patient-reported pain, function, satisfaction, or perceived recovery.

## Conclusion

In repeated observations from a single high-volume surgeon, the acetabular-only RATHA workflow was associated with lower perceived workload and a lower mean whole-case stress index than the CTHA workflow despite longer operative time. Whole-case RMSSD, heart rate, ventilatory parameters, and energy expenditure rate did not differ significantly between workflows. Phase-specific associations varied across operative steps, indicating that the between-technique differences were not uniform throughout the procedure. These hypothesis-generating within-operator findings warrant validation in multisurgeon studies examining different levels of surgical experience, robotic workflows, and operative contexts.

## Data Availability

The datasets generated and/or analyzed during the current study are available from the corresponding author on reasonable request.

## Abbreviations

ASA: American Society of Anesthesiologists
BMI: Body mass index
CI: Confidence interval
CTHA: Conventional total hip arthroplasty
DDH: Developmental dysplasia of the hip
FDR: False discovery rate
HOA: Hip osteoarthritis
HR: Heart rate
HRV: Heart rate variability
IQR: Interquartile range
MAKO: MAKO robotic-arm assisted system
MV: Minute ventilation
ONFH: Osteonecrosis of the femoral head
RATHA: Robot-assisted total hip arthroplasty
RMSSD: Root mean square of successive differences
RR: Respiratory rate
SD: Standard deviation
SURG-TLX: Surgery Task Load Index
THA: Total hip arthroplasty
TKA: Total knee arthroplasty
